# A Three-Dimensional Human Atrial Model with Fiber Orientation. Electrograms and Arrhythmic Activation Patterns Relationship

**DOI:** 10.1371/journal.pone.0050883

**Published:** 2013-02-11

**Authors:** Catalina Tobón, Carlos A. Ruiz-Villa, Elvio Heidenreich, Lucia Romero, Fernando Hornero, Javier Saiz

**Affiliations:** 1 Instituto Interuniversitario de Investigación en Bioingeniería y Tecnología Orientada al Ser Humano (I3BH), Universitat Politècnica de València, Valencia, Spain; 2 Departamento de Sistemas, Universidad de Caldas, Manizales, Caldas, Colombia; 3 Universidad Nacional de Lomas de Zamora, Buenos Aires, Argentina; 4 Servicio Cirugía Cardiaca, Hospital General de Valencia, Valencia, Spain; 5 Departamento de Informática y Computación, Universidad Nacional de Colombia Sede Manizales, Manizales, Caldas, Colombia; University of Cambridge, United Kingdom

## Abstract

The most common sustained cardiac arrhythmias in humans are atrial tachyarrhythmias, mainly atrial fibrillation. Areas of complex fractionated atrial electrograms and high dominant frequency have been proposed as critical regions for maintaining atrial fibrillation; however, there is a paucity of data on the relationship between the characteristics of electrograms and the propagation pattern underlying them. In this study, a realistic 3D computer model of the human atria has been developed to investigate this relationship. The model includes a realistic geometry with fiber orientation, anisotropic conductivity and electrophysiological heterogeneity. We simulated different tachyarrhythmic episodes applying both transient and continuous ectopic activity. Electrograms and their dominant frequency and organization index values were calculated over the entire atrial surface. Our simulations show electrograms with simple potentials, with little or no cycle length variations, narrow frequency peaks and high organization index values during stable and regular activity as the observed in atrial flutter, atrial tachycardia (except in areas of conduction block) and in areas closer to ectopic activity during focal atrial fibrillation. By contrast, cycle length variations and polymorphic electrograms with single, double and fragmented potentials were observed in areas of irregular and unstable activity during atrial fibrillation episodes. Our results also show: 1) electrograms with potentials without negative deflection related to spiral or curved wavefronts that pass over the recording point and move away, 2) potentials with a much greater proportion of positive deflection than negative in areas of wave collisions, 3) double potentials related with wave fragmentations or blocking lines and 4) fragmented electrograms associated with pivot points. Our model is the first human atrial model with realistic fiber orientation used to investigate the relationship between different atrial arrhythmic propagation patterns and the electrograms observed at more than 43000 points on the atrial surface.

## Introduction

The most common sustained cardiac arrhythmias in humans are related to the atria. Different atrial arrhythmias, mainly atrial fibrillation (AF), often provoke disabling symptoms and severe complications such as heart failure and stroke [1]. There are several experimental observations regarding the important role of the anatomical structure and electrophysiological heterogeneity on atrial electrical activity both in physiological and pathological conditions [2]–[4]. Additionally, it has been shown that atrial tachyarrhythmias produce a set of changes in atrial properties that lead to their perpetuation. These changes, denoted atrial remodeling, include alterations in the electrical cellular activity and in the anatomical structure. They have been described in several animal models [5] and in humans [6], [7]. Changes in electrical activity cause a decrease in refractoriness subsequent to a significant action potential duration (APD) shortening [6]–[8], which may help the initiation and maintenance of multiple reentrant waves, as suggested by experimental studies [5], [6].

It is well known that atrial tachyarrhythmias can be caused by various mechanisms, including rapid local ectopic activity, single-circuit reentry and multiple-circuit reentry [9]–[12]. It is important to know the fundamental mechanisms underlying atrial arrhythmias since they have implications in antiarrhythmic therapy. It is thought that different mechanisms lead to changes in the characteristics of spatiotemporal organization of atrial tachyarrhythmias. For the study of the spatiotemporal organization of atrial tachyarrhythmias, different signal analysis techniques are currently being used to analyze the electrograms (EGM) recorded at different points of the atrial surface. These include the analysis of EGM morphology [13]–[15], dominant frequency (DF) [16]–[21] and regularity [19] or organization index (OI) [22], [23].

Experimental and clinical studies have shown various degrees of spatiotemporal organization during sustained AF [10], [18], [24]. Recent evidence from high-density mapping and spectral analysis has shown that AF is associated with rotors and regular repetitive activation in a portion of the atrium. In addition, areas of complex fractionated atrial electrograms (CFAE) and high DF have been proposed as critical regions for maintaining AF [10], [24], [25]. Thus, identification of areas with these features may allow targeted ablation procedures to improve success rates. The OI, defined by Everett *et* *al* [22] for the quantification of the organization of atrial activity from spectral analysis of EGM is another parameter that is used to identify critical areas for maintaining atrial arrhythmias. However, there is a paucity of data on the relationship between CFAE, DF and OI. Even more, despite the important experimental and clinical evidence obtained in recent years, the relationship between the different characteristics of EGM and the propagation pattern that underlies them are still far from being completely understood.

In this study, a realistic three-dimensional (3D) computer model of the human atria is used to investigate the relationship between different atrial arrhythmic propagation patterns and the EGM observed at different points on the atrial surface. Firstly, an anatomically accurate 3D model of human atria was developed. Our model includes a realistic geometry with fiber orientation, anisotropic conductivity and electrophysiological heterogeneity for different atrial tissues. Then, different tachyarrhythmic episodes in the electrically remodeled model of human atria were simulated and the EGM were analyzed to characterize the arrhythmic patterns. This model is one of the most complete 3D human atrial models ever developed and it provides a useful tool for investigating the complex phenomena involved in atrial tachyarrhythmias by simulating experimental and clinical situations difficult to perform *in vitro* or *in vivo*.

## Methods

### 3D model of human atria

**Figure 1:**
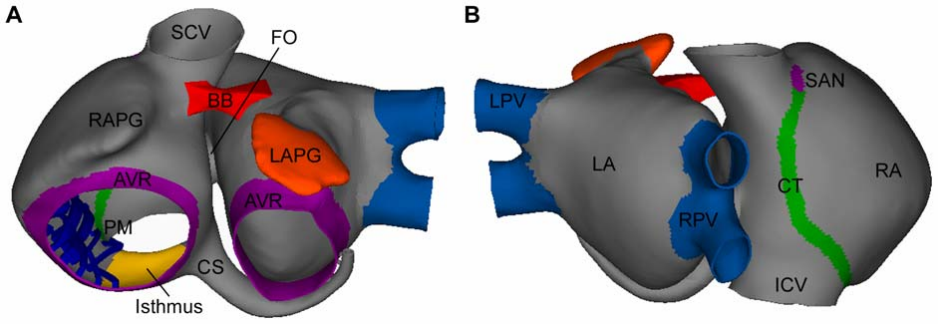
3D model of human atria. Frontal (A) and dorsal (B) views of the 3D model of human atria. Colored areas show regions with different conductivity and/or electrophysiological heterogeneity.

An anatomically realistic 3D model of human atria that includes fiber orientation was developed. Our model comprises the following main anatomical structures: left atrium (LA) and right atrium (RA), twenty pectinate muscles (PM) in the RA free wall, the fossa ovalis (FO) in the septum with its limbus, Bachmann's bundle (BB), the crista terminalis (CT), left and right appendages (LAPG and RAPG), left and right pulmonary veins (LPV and RPV), superior and inferior caval veins (SCV and ICV), the isthmus of RA, atrioventricular rings (AVR) and the coronary sinus (CS). The sinoatrial node (SAN) is situated near the ostium of the SCV. The CT layer originates next to the SAN and crosses the RA towards the ICV while the BB starts in a region next to the SCV and extends through the LA. The CS starts in the RA and extends along the posterior atrioventricular sulcus. The model also includes three different pathways for the inter-atrial conduction of electrical propagation: the BB, the limbus of the FO and discrete sites of the CS. Figure 1 shows the frontal (Figure 1A) and dorsal (Figure 1B) views of the model.

The original set of surfaces of the model was obtained from Harrild and Henriquez [26]. These surfaces were compared with data from the literature of normal human atrial structures and histological observations [2], [27], [28], and were modified. The dimensions of the different structures of the model are near the experimentally recorded average in human atria (see Table 1). The atrial wall is a monolayer surface, except the BB and the PM, which are solid structures.

**Table 1:** Dimensions of the structures in the model.

Structure [axes]	Model size (cm)	Reference
RA diameter [A-P/M-L/S-I]	4.5/4.3/4.2	28
LA diameter [A-P/M-L/S-I]	4.3/3.7/3.7	28
VP diameter [M-L/S-I]	0.9/1.1	27,28
ICV diameter [M-L/S-I]	2.2/2.0	55
SCV diameter [M-L/S-I]	1.9/2.1	28,55
MV diameter [M-L/S-I]	2.1/2.8	28
TV diameter [M-L/S-I]	2.9/2.8	28
CS orifice diameter	0.73	28,56
Isthmus [A-P/M-L]	2.4/1.9	56
LAPG [A-P]	5.5	57

A-P: ateroposterior; S-I: superorinferior; M-L: mediolateral; MV: mitral valve; TV: tricuspid valve.

### Fibers orientation and anisotropy

**Figure 2:**
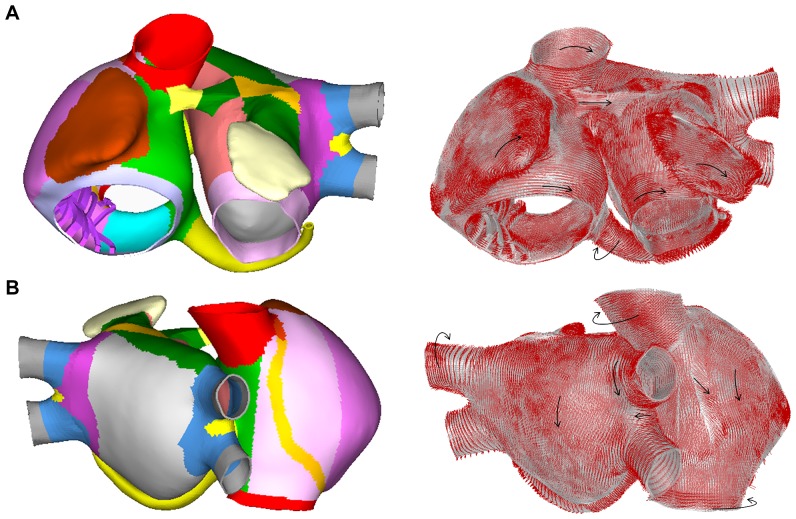
Fiber orientation in the model. Frontal (A) and dorsal (B) views of the model. The model was divided into 42 areas (represented with different colors) according to the orientation of the muscle bundles (left) and fiber orientation (see black arrows) assigned to the main areas (right).

One of the key characteristics of the atrial model is the incorporation of realistic fiber orientation based on histological observations [2], [27], [29]–[31]. Our model was divided into 42 areas according to the orientation of the main muscle bundles (circular, longitudinal, transverse or oblique) in order to assign a realistic fiber direction to each region. In Figure 2, the main areas of the model and their fiber orientation can be seen. It shows circulating muscle bundles [2], [27], [31] around the CS and orifices of the pulmonary veins (PV), the SCV and ICV, the AVR, and both appendages (LAPG and RAPG). Fibers of BB, CT and PM have a longitudinal orientation. The free walls of both atria (RA and LA) are smooth and primarily have vertical fibers. The right free wall includes the CT, a fibrous bundle that runs vertically between both caval veins. The fibers between the superior and inferior PV are horizontally positioned, whereas the ostium of the PV contains a complex arrangement of vertical, horizontal and circular fibers [30]. This model is an improved version of the atrial models previously developed by our group [32]–[34].

### Electrophysiological model

Nygren's model [35] of the human atrial action potential (AP) was implemented to reproduce the cellular electrical activity. In this study, the electrophysiological heterogeneity was introduced to reproduce APs in different parts of the atria [36]: CT, PM, APG, AVR and AWM (atrial working myocardium), which includes the remaining atrial structures. The maximum conductance values of I_t_, I_Kr_ and I_CaL_ were modified in Nygren's cellular model to obtain different AP models (see control values in Table 2).

**Table 2:** Conductances and APD_90_ of isolated cells in the regions of the model.

	Condition	CT	PM	APG	AVR	AWM
***g*** **_t,max_**	Control	10.6	8.3	4.2	8.3	7.5
	Remodeled	1.6	1.2	0.6	1.2	1.1
***g*** **_CaL,max_**	Control	10.3	6.2	6.7	4.2	6.7
	Remodeled	2.7	1.6	1.7	1.1	1.7
***g*** **_Kr,max_**	Control	0.4	0.4	2.3	2.3	0.5
	Remodeled	0.4	0.4	2.3	2.3	0.5
***g*** **_K1,max_**	Control	4.2	4.2	4.2	4.2	4.2
	Remodeled	10.5	10.5	10.5	10.5	10.5
**APD_90_ (ms)**	Control	307	237	245	180	282
	Remodeled	92	73	78	56	80

*g*_t,max_, *g*_CaL,max_, *g*_Kr,max_, *g*_K1,max_ *:* the maximum conductance of I_t_, I_CaL_, I_Kr_ and I_K1_ currents, respectively.

The basic equation to calculate transmembrane voltage V_m_ is:
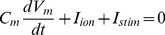
(1)where C_m_ is the specific membrane capacitance (50 pF), I_ion_ is the total membrane ionic current, V_m_ is the membrane potential and I_stim_ is the stimulus current.

To reproduce atrial electrical remodeling, changes in the maximum conductance and kinetics of different ionic channels of human atrial cells observed in experimental studies of chronic AF [7] have been incorporated into the AP models. The following parameters were altered [37]: maximum conductance of I_K1_ was increased by 250% while the maximum conductance values of I_CaL_ and I_t_ were decreased by 74% and by 85%, respectively (see remodeled values in Table 2), the time constant of the fast inactivation of I_CaL_ was increased by 62%, the activation curve of I_t_ was shifted by +16 mV, and the inactivation curve of I_Na_ was shifted by +1.6 mV.

**Figure 3:**
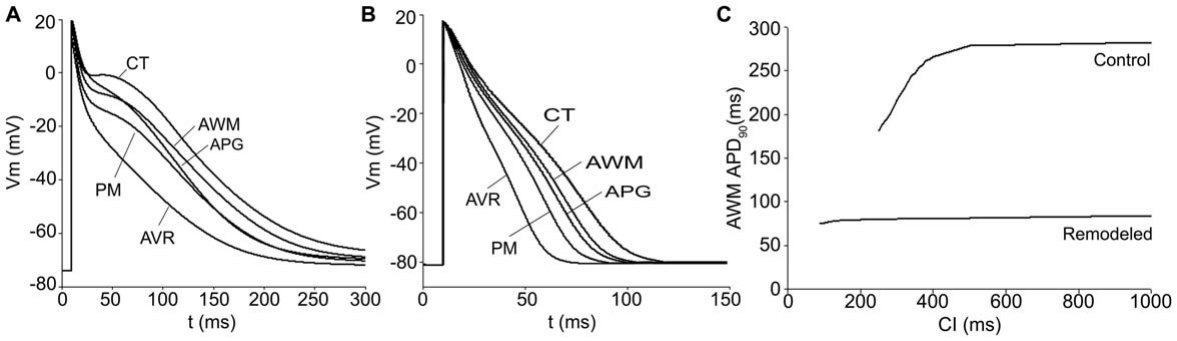
AP for different atrial areas and APD_90_ restitution curve for AWM under physiological and remodeling conditions. AP time courses for the considered atrial cellular models (CT, PM, APG, AVR and AWM) under physiological (A) and remodeling conditions (B). APD_90_ restitution curve for AWM under physiological (control) and remodeling conditions (C).

Figure 3 depicts the APs for the different atrial cellular models considered, under both physiological (control) and remodeling conditions (Figure 3A and 3B, respectively). In these figures, we present the last AP obtained when a train of 10 stimuli at a basic cycle length of 1000 ms was applied. The corresponding APD_90_ (APD to 90% of repolarization) values for the different atrial cells (both for control and remodeling conditions) are shown in Table 2. Under control conditions, APD_90_ showed high values (ranged from 180 ms to 307 ms) in agreement with experimental data [7], [36] and a great APD dispersion was observed (APD_90_max – APD_90_min  = 127 ms). By contrast, under remodeling conditions, the APD decreased (ranged from 56 ms to 92 ms) and a smaller APD dispersion was observed (APD_90_max – APD_90_min  = 36 ms). Figure 3C shows the APD_90_ restitution curve for control and remodeled cells when the coupling interval (CI) between pulses is increased. Remodeling conditions not only induce a shortening in the APD_90_ but also reduce the frequency dependent adaptation of APD_90_.

### Action potential propagation and electrograms

In this study, the monodomain model of the electrical propagation of AP along the tissue model is described by the following reaction-diffusion equation [13], [38], [39]:
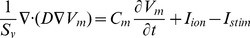
(2)where *S_v_* corresponds to the surface-to-volume ratio and *D* is the conductivity tensor. The monodomain equation was solved using a finite element method [40].

Our model includes regions with very high (CT), high (BB, limbus of the FO and PM), low (PV and isthmus of the RA) and very low conductivity (SAN). The AWM has mid-range conductivity (see areas with different colors in Figure 1).

A simplified two-dimensional model of 150×150 cells was used in order to select the values of tissue conductivity in the longitudinal and transversal direction of the fibers for the different regions. The conductivity values were fixed in order to obtain conduction velocities within the ranges reported in the literature [41]–[45], from 25 cm/s in very slow regions (SAN) to 143 cm/s in very fast regions (CT bundles) in normal (without remodeling) atria (see Table 3). Table 3 also shows that atrial remodeling provokes a reduction in the conduction velocities, between 9% and 24%. Anisotropic conduction was also assumed with a transversal to longitudinal ratio of conductivity of 1∶9 for CT, which is in accordance with experimental studies [44] that have reported an anisotropy ratio in CT of 1∶10. The isthmus of the RA and the SAN were set as isotropic, while an anisotropic ratio of 1∶2 was used for BB, limbus of FO, PV and AWM [46].

**Table 3:** Conductivities and conduction velocities in the regions of the model.

	CT	BB, PM, and FO rim	PV	Isthmus	SAN	AWM
**Longitudinal conductivity (S/m)**	0.7	0.5	0.15	0.1	0.05	0.2
**CV in control (cm/s)**	143	120	54	44	25	69
**CV in remodeled (cm/s)**	130	107	46	37	19	61

CV: conduction velocity.

Unipolar EGM at more than 43000 points of the atria surface (0.2 mm from the surface) were simulated. The extracellular potential (Фe) was computed using the large volume conductor approximation [38], [47], [48]:
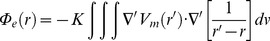
(3)where ∇'V_m_ is the spatial gradient of transmembrane potential V_m_, K is a constant that includes the ratio of intracellular and extracellular conductivities, r is the distance from the source point (x, y, z) to the measuring point (x', y', z') and dv is the differential volume.

Surface pseudo-unipolar EGM at different points were visually inspected in order to seek their morphologies as either single, double or fractionated potentials. Double potentials were defined as EGM with two negative deflections, and CFAE were defined as EGM either exhibiting multiple (more than two) deflections or EGM with continuous (more than 50 ms) electrical activity without an isoelectric line [49]. EGM were digitally processed (every millisecond) with a 40–250 Hz band-pass filter, then rectified and low-pass filtered at 20 Hz [50]. Next, spectral analysis of the signals was performed with a fast Fourier transform obtaining a spectral resolution of 0.12 Hz. The DF defined as the frequency corresponding to the highest peak of the power spectrum was calculated. In addition, to measure the periodicity of the signal and the variability of the frequency in the spectrum, the OI was also calculated. The spectral power of the DF and its three harmonic peaks were calculated by computing the area under their peaks over a 0.75 Hz window. Finally, the OI was obtained as the ratio of this spectral power to the total power of the spectrum [23], [50].

### Numerical and computational methods

A hexahedral mesh was built from the three-dimensional anatomical model using Femap from Siemens PLM software. This mesh includes 52906 elements and 100554 nodes with a spatial resolution ranging from 300 to 700 μm, the average element size being approximately 530 μm. Similar spatial resolutions have been used in previous computational studies of atrial propagation [26], [51]. Equation (2) was numerically solved using the software EMOS [52], [53]. EMOS is a parallel code (mpi-based) that implements the finite element method (FEM) [40] and the Operator Splitting [54] for solving the monodomain model (Eq. 2). The time step was fixed to 0.02 ms. Simulation of 10 seconds of atrial activity took 14 hours on a computing node with two 6-core Intel Xeon X5650 clocked at 2.66 GHz and 48GB DDR3 RAM.

### Simulation protocols

SAN and ectopic stimulation were simulated by applying rectangular current pulses of 6 ms in duration and 30 μA in amplitude on an area of approximately 10 mm^2^. Atrial tachyarrhythmias were generated by the S1–S2 protocol, which consisted of a train of stimuli with a basic cycle length of 1000 ms applied during 10 seconds in the SAN area to simulate the sinus rhythm (S1). After the last beat of the SAN stimulation, an ectopic focus (S2) was delivered in six different locations in the atria: CT, SCV, isthmus of RA (near the CS), and ostium of the LPV and of the RPV. Two different ectopic foci S2 were used in our study: 1) a train of six stimuli at cycle length (CL) of 130 ms; and 2) a continuous focus also at CL of 130 ms.

## Results

### Atrial action potential propagation

**Figure 4:**
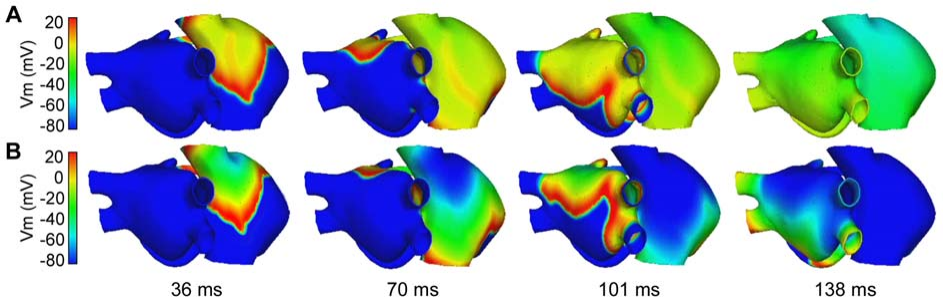
Sinus rhythm propagation under physiological and remodeling conditions. Propagation of the last sinus beat applied for both physiological (A) and remodeling (B) conditions. The color scale represents the range of AP values (mV). The depolarizing fronts can be identified by the red color.

An atrial propagation pattern in sinus rhythm was simulated in the model by applying a periodic stimulus (10 beats) at a basic cycle length of 1000 ms in the anatomic location of the SAN. Figure 4 shows different snapshots of the propagation of the last beat applied for both physiological (Figure 4A) and remodeling (Figure 4B) conditions. A delayed activation front in the remodeled atria can be observed. In Figure 4, it is possible to note how the stimulus applied in the SAN region caused the initiation of a propagating wavefront that quickly spread to the ICV favored by the high conductivity of the CT arch. The anisotropy of the CT caused an almost triangular wavefront (see snapshots at 36 ms in Figures 4A and 4B). The depolarizing wave reached the BB after 22 ms and propagated to the anterior septal portion of the LA through interatrial BB, inducing the first activation of the LA at 46 ms (in normal atria) and 54 ms (in remodeled atria) after the SAN activation. Thereafter, at 53 ms and 62 ms from the SAN activation for normal and remodeled atria, respectively, the interatrial connection at the limbus of the FO contributed to left atrial septal activation. The activation wave also propagated to LA through the third interatrial connection, the RA-CS-LA pathway, at 87 ms and 98 ms, respectively, and the first LA inferior wall activation was observed at 117 ms and 132 ms for normal and remodeled atria, respectively.

In Figure 4 (see snapshots at 70 and 101 ms) the delay in the propagation induced by remodeling conditions can be observed. The entire atrial depolarization occurred at 120 ms in normal conditions and at 138 ms in remodeled atria (see snapshot at 138 ms in Figure 4B), finishing in the distal left APG. Moreover, the whole atrium repolarized faster in remodeling conditions than under physiological conditions, at 234 ms and 432 ms after SAN activation, respectively.

After the 10^th^ stimulus was applied in the SAN zone, two different ectopic foci (S2) were applied at six different points of the atria (see methods): 1) a burst of six beats, and 2) a continuous ectopic focus, both with a CL of 130 ms. When we applied the burst of six beats, we observed either atrial flutter (AFL) or reentrant tachycardia in five of the six locations tested (isthmus of RA near the CS, SCV, CT, ostium of the LPV and center of the LA posterior wall), and AF when the ectopic focus was applied in the ostium of the RPV. Different AF patterns were also observed when a continuous ectopic focus was applied in the six locations.

### Atrial flutter and reentrant tachycardia

**Figure 5:**
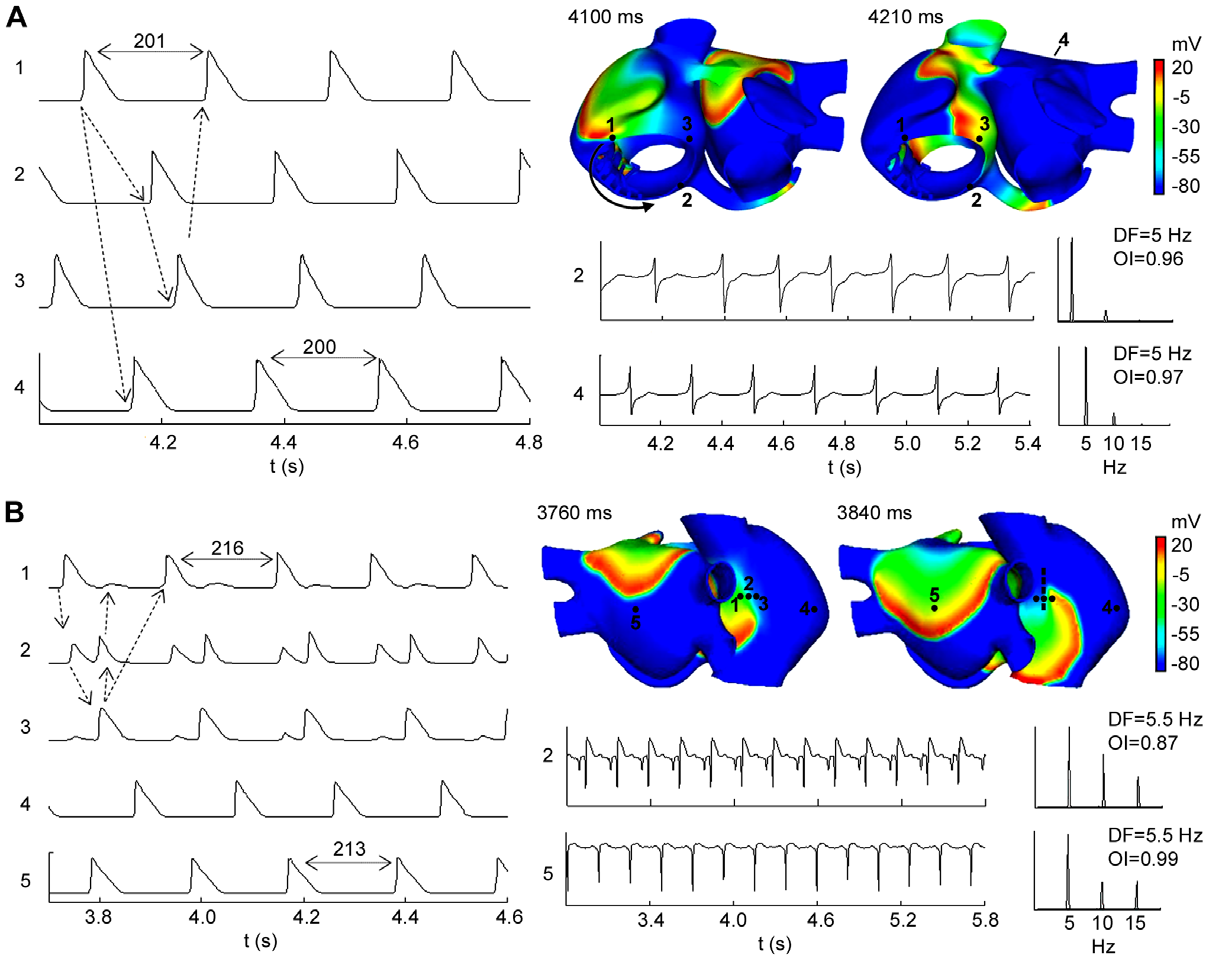
Flutter and reentrant tachycardia episodes. Snapshots, APs, EGM and spectral analysis in selected points. (A) Atrial flutter and (B) reentrant tachycardia episodes. In the snapshots, the color scale represents the range of values of the AP in mV. The depolarizing front is identified by the red color. The black arrow indicates the wavefront direction and the dash line a blocking line. AP time-courses of selected sites (indicated in the snapshots) are showed at the left, the dotted arrows indicate the activation sequence. EGM and their spectral analysis showing DF and OI values are shown (See text for more details).

Figure 5A depicts an example of AFL obtained by applying the burst of six ectopic beats in the isthmus of RA (near the CS), 231 ms after the last sinus stimulus. When the burst was applied, a conduction block was observed in the CS area which induced a macroreentry rotating counterclockwise around the tricuspid annulus (see the arrow in snapshot at 4100 ms in Figure 5A), that was maintained during the whole simulation (≈8 seconds). The activation pattern of this macroreentry resembles an AFL (see video S1 in the Supporting Information). The direction of the reentrant circuit can also be observed when comparing the APs of the sites 1, 2 and 3 in Figure 5A (see dotted arrows). The macrorentrant wavefront depolarized the rest of the atria, including the posterior wall of the LA (see site 4 in Figure 5A) with a 1∶1 activation pattern. The CL of this arrhythmic pattern was almost constant (≈200 ms) in the whole atria, a characteristic of AFL. The EGM in different points of the atria (see sites 2 and 4 in Figure 5A) only displayed single potentials, showing a stable and regular atrial activation, which is also characteristic of AFL. This regularity is reflected as a single narrow DF peak of 5.0 Hz and high OI values, close to unity, in sites 2 and 4. Similar DF and OI values were observed in the entire atrial tissue.

Figure 5B shows an example of reentrant tachycardia (see snapshots at 3760 ms and 3840 ms) obtained by applying a burst of six ectopic beats approximately in the middle of the CT, 160 ms after the last sinus stimulus. In this case, the ectopic focus initiated a reentry anchored to the RA, that propagated between the center and the top areas of the CT (see video S2 in the Supporting Information). This propagation pattern was favored by the greater anisotropy, fiber orientation and heterogeneity in the CT region. In fact, the CT area acts as a blocking line (see the dashed line in snapshot at 3840 ms in Figure 5B), maintaining the reentry activity and keeping the rest of the tissue in a reentrant tachycardia pattern. The blocking line is a source of electrotonic APs where functional conduction blocks are observed. The APs during tachycardia at five sites are also shown in Figure 5B. It is important to note that in the CT region, where the reentry turns, two impulses arrive to site 2 from sites 1 and 3 in each cycle. Site 3 shows electrotonic APs when the reentry passes through the region of site 1 and 2, but it is blocked before arriving to site 3. Likewise, site 1 shows electrotonic APs when the reentry turns and passes through site 3 and 2, but it is blocked before arriving to site 1. This reentrant activity spreads to the rest of the atria (see APs at sites 4 and 5 in Figure 5B).

The EGM observed in areas of conduction-block (see EGM at site 2) are characterized by double potentials, one short negative component associated with the electrotonic APs elicited when the reentry is passing at a close distance, and another larger and steeper negative component generated when the reentry passing through the electrode area. The EGM at site 5 displays single potentials with only negative deflections of short-time when the curved front passes on this site (see EGM at site 5 in Figure 5B). The EGM observed in the rest of the atria (out of the blocking line area) showed single potentials.

Only slight variations in the CL of APs were observed between different points, which suggest a periodic and regular reentrant activity. The entire atrial tissue presents similar DF (around 5.5 Hz) and high OI values (see sites 2 and 5 in Figure 5B, with values of 0.87 and 0.99, respectively). Reentrant tachycardias were also observed when the ectopic focus was applied in SCV, LPV and the LA posterior wall (data not shown).

### Atrial fibrillation

**Figure 6:**
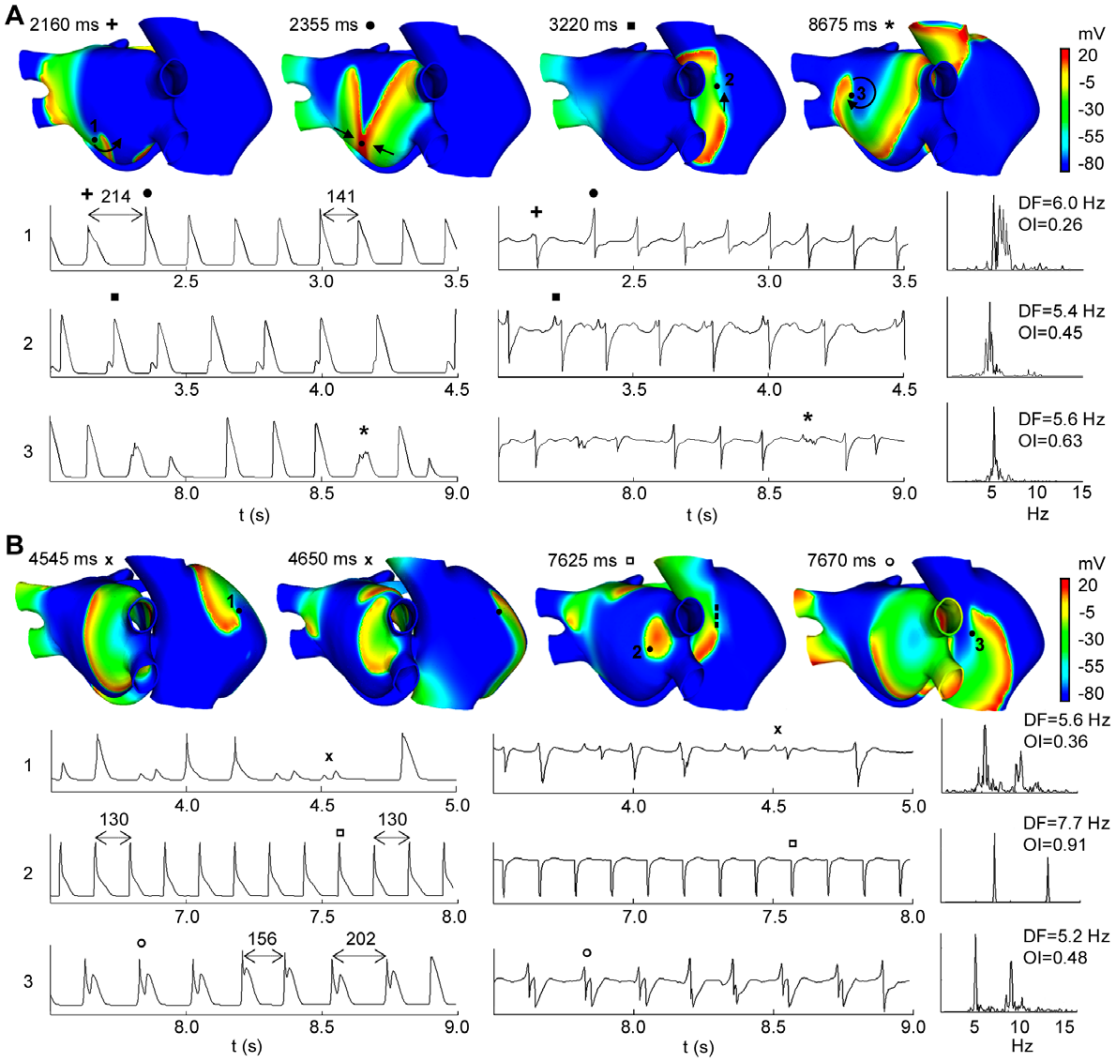
AF episodes. Snapshots, APs, EGM and spectral analysis in selected spoints. (A) AF triggered by a transient ectopic focus and (B) AF triggered by a continuous ectopic focus, both in the ostium of the RPV. The color scale represents the range of values of the AP in mV. The depolarizing front is identified by the red color. The black arrows indicate the direction of the wavefronts and the dash line shows a blocking line. AP time-courses of selected sites (indicated in the snapshots) are showed at left, down to the snapshots. The corresponding EGM and their spectral analysis showing DF and OI values are shown at the right. In (A): + indicates AP and single potential with only a long-lasting negative deflection (snapshot at 2160 ms), • indicates AP and single potential with positive deflection greater than negative (snapshot at 2355 ms), ▪ indicates AP and potential with a small additional positive deflection (snapshot at 3220 ms) and * indicates AP and CFAE (snapshot at 8675 ms). In (B): x indicates AP and potential with deviations from the baseline, CFAE, at site 1 (snapshots at 4545 ms and 4650 ms), □ indicates AP and potentials with only negative deflections at site 2 (snapshot at 7625 ms), and ○ indicates AP and double potentials at site 3 (snapshots at 7625 ms and 7670 ms).

Figure 6 shows two AF episodes triggered by an ectopic focus placed in the ostium of the RPV. In the first case, a train of six ectopic beats (CL = 130 ms) provoked an AF episode that was maintained by different reentrant mechanisms (see snapshots in Figure 6A). Indeed, rotors, figure of eight reentries, macrorreentries, fragmentations and wavefront collisions were observed in both atria (see video S3 in the Supporting Information). This irregular activity caused the appearance of electrotonic APs and irregular APs in different areas and at different times (see APs at sites 2 and 3 in Figure 6A).

The EGM primarily show irregular and polymorphic potentials, but double potentials and CFAE were also observed in minor areas. The first potential of the EGM calculated at site 1 (see + in EGM at site 1 in Figure 6A) only shows a long-lasting negative deflection when a spiral wavefront passes over this point and away from it (see the black narrow in snapshot at 2160 ms in Figure 6A). At this site, potentials with positive deflection greater than negative ones (see • in EGM at site 1 in Figure 6A) were observed when wave collisions occurred on the recording point (see snapshot at 2355 ms in Figure 6A).

The EGM calculated at site 2 shows potentials with a small additional positive deflection generated when the wavefront is fragmented (see ▪ in EGM at site 2 in Figure 6A). Then one of the two fronts is shifted to the top of the CT returning to depolarize the site 2 (see the arrow in snapshot at 3220 ms in Figure 6A), which gives rise to potentials with double positive deflections.

CFAE at site 3 were observed when the tip of the rotor turned on this point (see the arrow in snapshot at 8675 ms in Figure 6A). The wavefront surrounds the pivot point, without depolarizing it completely, which results in multiple low amplitude deflections in the EGM (see * in EGM at site 3 in Figure 6A).

Variations in the CL of the APs were observed between different points of the atria, which exceeded 20 ms in many cases. This fact is reflected in the appearance of multiple frequency peaks and low OI values in the spectral analysis. Heterogeneity in DF values and low OI values were observed in the entire atrial tissue (see spectral analysis of EGM at sites 1, 2 and 3 in Figure 6A).

Figure 6B shows an example of a focal AF episode triggered by a continuous ectopic focus applied in the ostium of the RPV. This ectopic activity also induced multiple reentrant waves that collided and fragmented with each other (see snapshots in Figure 6B and video S4 in the Supporting Information). Rotors with irregular trajectories that collided and fragmented creating new activation fronts were mainly observed in the RA. Although LA also presented fragmentation and collisions in the superior and anterior walls, the posterior wall near RPV was activated at a higher frequency by the wavefront generated by the ectopic focus.

In site 1 of Figure 6B, a reentry turns around a functional block for several milliseconds. Electrotonic APs reflected as deviations from the baseline at the EGM, CFAE, (see x in EGM at site 1 in Figure 6B) were observed when the reentry turned very close to site 1 (see snapshots at 4545 ms and 4650 ms in Figure 6B). In other instants, the spiral wavefront moved over the site 1 generating potentials with a single negative deflection in the EGM (snapshots not shown).

The APs and the EGM calculated in site 2, near the ectopic focus, show a rapid and regular atrial activation with similar CL values. The EGM has potentials with only negative deflections when the curved wavefront passes through this point (see snapshot at 7625 ms and □ in EGM at site 2 in Figure 6B). The stable and regular activity in this site is reflected in the spectral analysis as a single narrow DF peak of 7.7 Hz (value of the ectopic focus frequency) and high OI value close to unity.

Site 3 in Figure 6B shows APs and EGM for a point on the blocking line, which induced reentry anchored to the CT in certain instants (see the dashed line in snapshot at 7625 ms in Figure 6B). The reentry propagates between the center and the top areas of the CT, similarly to that observed during reentrant tachycardia in Figure 5B. Site 3 is depolarized by the wavefront and it is again activated, before repolarizing, by the front turning on line blocking. This behavior is reflected in EGM with double potentials (see ○ in EGM at site 3 in Figure 6B).

The only zone of the atria that shows a high frequency of activation (similar to the focus) and high OI values is the region of the posterior wall of LA near to the ectopic focus. The remaining tissue is unable to depolarize at this high frequency and it shows irregular CL, which is reflected in the spectral analysis with multiple frequency peaks and lower DF and OI values (DF of 5.6 Hz and OI of 0.36, and DF of 5.2 Hz and OI of 0.48, at sites 1 and 2, respectively, in Figure 6B). Every AF rhythms simulated when the continuous focus was applied in the other five locations (not shown) displayed stable and regular activity near the area of the ectopic focus and high DF values similar to the focus frequency. In the rest of the tissue, multiple reentrant waves generating irregular activity were observed.

## Discussion

### Validation of 3D model of human atria with complete fiber orientation

A 3D model of the human atria with realistic anatomy and fiber orientation was developed. Our model also includes real anisotropy characteristics and electrophysiological heterogeneities in the main atrial structures. To our knowledge, this model is one of the most complete 3D human atrial models ever developed.

Our model is based on the surfaces provided by Harrild and Henriquez [26] that were improved using detailed anatomical and histological observations [2], [27]–[31], [55]–[58]. We modified the geometry and dimensions of the PV, APG, PM and AVR rings, and the CS was created, in accordance with human data [27], [28], [55]–[58]. Specifically, the diameters antero-posterior and medial-lateral of our model are 4.5 cm and 4.3 cm for the RA, respectively, and 4.3 cm and 3.7 cm for the LA, respectively. These values fall within the ranges reported by Cohen *et* *al* [28] in humans (2.8–5.2 cm and 2.9–5.3 cm for RA; and 2.0–5.2 cm and 2.4–5.2 cm for LA). In addition, the diameters for PV (1.1 cm), CS (0.7 cm), SCV (1.9 cm), mitral valve (2.1 cm) and tricuspid valve (2.9 cm) are also within the ranges reported by Cohen *et* *al* (0.7–1.6 cm for PV, 0.4–1.0 cm for CS, 0.8–2.0 cm for SCV, 2.0–3.8 cm for mitral valve, and 2.0–4.0 cm for tricuspid valve).

Our model also includes anisotropy and fiber orientation. It has been observed that atrial anatomy, anisotropy and fiber orientation are critically important in determining the spread and direction of the activating wave front [2], [3], [4], [29], [31]. There are a number of well-defined bundles in the atrial muscle where the AP propagates faster [29], [41]–[43]. These bundles include the CT, BB, the PM and the limbus of the FO. All of them are considered in our model. The atrial model was divided into 42 regions and a detailed fiber orientation was included in each region based on histological observations [2], [27]–[31], [59], [60]. This model goes beyond previous models, as it not only considers a detailed atrial anatomy, regional heterogeneity and anisotropy, but also accurate fiber orientation for the whole atria. Jacquement *et al* [13] developed an atrial model of simple geometry with a coarse fiber structure, which was manually introduced. Seeman *et al* [61] and recently Aslanidi *et al* [62] included only the fiber orientation of the main bundles (BB, PM and CT) in their human atrial model. Krogh-Madsen et al implemented the Harrild and Herriquez human atrial model [26] without structural modifications, anisotropy and fiber orientation to simulate arrhythmic episodes [63]. Ho *et al* [31] argued that the heterogeneous myoarchitecture of RA and LA and the inter-atrial septum must be considered in computer models that seek to investigate mechanisms of atrial arrhythmias, as our model does. An image-based anatomical model of the sheep atrial fiber orientation has been recently presented [64]. The model reproduces the whole atria with highly detailed myofiber architecture while our model simplifies the complex myocardial structure by dividing the whole atria into 42 regions. Fiber orientation in both models has some similarities, for example fibers at the base of SVC and around PV have circumferential orientation or highly organized tracts in the CT and PM. However, it is not clear how similar are fiber architecture in the whole atria between sheets and human.

Our model also includes a realistic RA-CS-LA interatrial connection; where the CS connects electrically the RA and the LA through some fiber tracks. Different anatomic studies [65] have shown that the CS musculature is continuous with LA myocardium in the proximal portion of the CS and with RA myocardium at the CS orifice. Therefore, the CS musculature may form an electrical connection between the RA and the LA.

The electrophysiological heterogeneity also plays an important role during atrial conduction. In order to reproduce the AP morphologies in different parts of the atria, the human atrial model developed by Nygren was modified based on the electrophysiological heterogeneity observed in canine atria [36] as previously suggested [61]. The effect of electrical remodeling was introduced by altering different currents (I_K1_, I_CaL_, I_t_ and I_Na_) in accordance with experimental studies of chronic AF [7]. In this study, under normal conditions, AP showed morphological differences in the different electrophysiological zones, consistent with those reported experimentally [36]. It is noteworthy that, under electrical remodeling conditions, the APs showed similar morphologies for different atrial zones with only slight APD variations between them.

#### Atrial action potential propagation

Our model reproduces the normal atrial activation originated in the SAN, both under physiological and remodeling conditions. Under physiological conditions, the activation initiated in the SAN region reached the BB after 22 ms, which follows experimental studies that have reported the first activation of BB at approximately 19 ms [59]. The propagating wavefront quickly spreads to the ICV, becoming almost triangular, favored by the high conductivity of the longitudinal fiber orientation of the CT. These characteristics of the wavefronts have been reported for normal sinus rhythm in humans [43].

In our simulation study, the RA free wall was completely depolarized after 60 ms. This result is in agreement with experimental observations of the complete RA wall activation at 57 ms [43]. Interestingly, the LA was also activated through the limbus of FO at 53 ms, and through the RA-CS-LA connection at 117 ms, which is consistent with recent studies that have suggested that the activation of the posterior epicardial LA takes place at 54±10 ms [66] and that a third interatrial electrical connection exists in the region of the CS [65], [66].

The complete LA activation was observed in the distal LAPG at 120 ms, which is also in agreement with experimental results. Canavan *et al* [67] showed that the last activation in the atrial tissue occurs just before 120 ms. Lemery *et al* [66] reported the latest LA activation at 116±18 ms. In addition, it has also been documented that atrial activation ends at the LAPG [68].

Under remodeling conditions, the latest atrial activation also occurs in our model in the distal LAPG, but after 138 ms, showing a conduction delay. The delay is due to an overall reduction of the conduction velocity that changes from 9% to 24% depending on the zone [7], [69]. Experimental studies in dogs [70] observed a mean reduction of 25% in conduction velocity values measured in remodeling conditions. Even more, under remodeling conditions a faster repolarization of the whole atria was observed due to a reduced APD.

Simulated tachyarrhythmic episodes resulted from the application of ectopic foci with both transient and continuous activity. It has been demonstrated that extrastimuli, either both transitory or continuous, can act as triggers and, in some cases, they may be responsible for the initiation and maintenance of AF episodes [9], [71]. In our study, the foci were applied in six different locations in the atria: CT, SCV, isthmus of RA (near the CS), center of the LA posterior wall and ostium of the LPV and RPV. Although tachyarrhythmic episodes are initiated by focal triggers most commonly localized in areas near the PV [9], [71], ectopic foci have also been recorded in other regions. In an extensive cases report [72] the ectopic foci were located in the posterior wall of the LA including VP (38.3%), in the SCV (37%), in the CT (3.7%) in the ligament of Marshall (8.2%), in the CS (1.4%) and in the interatrial septum (1.4%).

In our simulations, one AFL, four reentrant tachycardias and one AF episode were obtained by applying transient ectopic foci, but only AF episodes were observed when a continuous ectopic focus was applied in the same locations.

### Flutter and reentrant tachycardia

The application of the train of six ectopic beats in the isthmus of RA (near the CS) triggered an episode of AFL. It was maintained by a macroreentry located in the RA, turning counterclockwise around the tricuspid annulus. In patients, it has been observed that a high percentage (85%) of AFL is due to reentrant excitation travelling around the tricuspid valve ring [73]. Notably, our model can reproduce this kind of AFL episode, where the circular fiber orientation around the tricuspid annulus (as in the real atrial musculature) plays an important role in the AFL reentrant circuit. The macroreentrant circuit activated the rest of the atria with a 1∶1 pattern of activation and functional block was not found in the CT. This supports similar observations reached in a previous analysis of human AFL [74], where the atrial activation in the right lateral wall was uniform, without blocking in the region of the CT. In our simulation of AFL, no variation was observed in the CL (≈200 ms) in the different regions of the atria. This result is also consistent with the mean values of CL (238 ms and 245 ms) obtained in experimental and clinical studies [75], [76].

The EGM recorded in different points of the atria presented high organization, with uniform and regular single potentials, which reflected in the spectral analysis as a single narrow DF peak. This is in agreement with EGM recorded *in vivo* [77] in both LA and RA which also showed uniform and regular potentials. In our model, the spectral analysis showed DF of 5 Hz during AFL, which falls within the range reported in humans (4.2–5.8 Hz) [78]. Additionally, EGM also showed high OI values according to the high regularity in the atrial activation [23].

Application of the ectopic focus in the CT, SCV, ostium of LPV and the center of the LA posterior wall triggered reentrant tachycardia episodes. When the ectopic focus was applied in the CT, the reentrant tachycardia was maintained by a reentry anchored to the CT. The reentry moved between the central and the superior part of the CT, showing a block line in this region. Experimental studies have shown a great number of the reentrant tachycardias in absence of structural heart diseases occurring along the CT [60], [79], due to the anisotropy in this region that favors the role of CT as a natural barrier to the atrial activation. Even more, it has also been demonstrated in experimental and clinical studies that the CT is an anatomical substrate underlying atrial arrhythmias [74], [80].

The reentrant tachycardia produced EGM with mostly single, uniform and regular potentials, linked to stable and regular atrial activation. This organized activity is reflected in the spectral analysis with a single narrow DF peak (consistent with the CL of atrial tachycardia) and a high OI value in most areas of the atria. A 1∶1 uniform activation pattern across both atria and a high regularity in the atrial activation were observed. Experimental and clinical studies [77], [79] have shown stable and regular atrial activation with mostly single and regular EGM during reentrant tachycardias. Everett *et* *al* [23] have linked high OI values with high regularity in atrial activation. It is noteworthy that double potentials were observed in the EGM along the CT when it acts as a blocking line. Interestingly, our results also confirm that double potentials (or at least part of them) could be related to electrotonic potentials provoked by sequential activation on both sides of the blocking line. In an experimental study on atrial EGM during AF, Konings *et* *al* [15] registered double potentials along the lines of conduction block, similar to the results of our simulations.

### Atrial fibrillation

When a train of six ectopic beats in the ostium of the RVP was applied, an episode of AF was initiated (Figure 6A). Multiple reentrant waves, rotors, fragmented fronts and collisions were observed, which are characteristic of fibrillatory activity [10], [15], [77]. CL variations and EGM with single potentials double potentials and CFAE were observed. Experimental and clinical studies of AF [16], [17], [77] have recorded intracardiac polymorphic EGM, and irregular and disorganized activation patterns in areas with fibrillatory conduction.

In our study, EGM showed only a long-lasting negative deflection when a spiral wavefront passed over the recording point, and potentials with positive deflection greater than the negative ones when wave collisions occurred. These results are consistent with previous studies [13], [81] which have shown that the EGM morphology is related to the shape of the wavefront and to its curvature, suggesting that single potentials may have different morphologies. Our results confirm the results obtained by Jacquemet *et al* [13] in simulation studies using a model with simplified fiber structure. He observed potentials with a positive deflection much lower than the negative deflection during either curved or spiral fronts. He also obtained potentials with a much larger positive deflection than negative deflection during a collision between two wavefronts. However, Konings *et al* [15] reported short-double potentials along either side of the line of collision, unlike observations in our study. The differences could be due to the collisions between several wavefronts (>2) observed by Konings *et al*, whereas in our study the collisions occur mainly between two wavefronts.

In our study, CFAE were observed when the tip of the rotors turned on the recording point (pivot point). During the last decades experimental and clinical studies have demonstrated that the maintenance of the AF in many cases depends on these rotors [17], [82], [83]. Recent observational studies have demonstrated that substrates serving as ‘‘AF perpetuators’’ can be identified by searching for areas that have CFAE, but the underlying etiology of CFAE and their relationship with rotors has not yet been elucidated [84]. Konings *et al* [15] recorded CFAE during AF in humans, suggesting that CFAE can indicate pivot points, slow conduction and complex reentrant patterns. Zlochiver *et al* [14] demonstrated that rotor meandering might also underlie, at least in part, the CFAE that they observed close to the driver. Our results reveal a direct relationship between the tip of the rotor (pivot point) and the CFAE.

The spectral analyses display multiple frequency peaks around the DF peaks and lower OI values than those observed during either reentrant tachycardia or AFL. These results are in agreement with experimental studies [15]–[17], [77], reporting that spectral analysis of the EGM recorded during AF episodes shows multiple frequency peaks in areas with irregular and unstable activation. Indeed, conduction blocks and wave collisions increase the irregularity and variability in the frequency [15], [16], [82], [85]. Everett *et* *al* [23] associated low OI values with the presence of EGM with double and fragmented potentials in canine models of AF.

In our study, the focal AF triggered by an ectopic focus with continuous activity between RPV showed substantial differences from the previous AF. Nearby zones and the location of the ectopic focus showed APs and EGM with rapid and regular activation with single potentials and similar CL values between them. This electrical activity gives rise to a single frequency peak with the highest DF value, similar to the frequency of the ectopic focus and with the highest OI values, in accordance with experimental observations. Lin *et* *al* [16] have reported that EGM recorded near the ectopic sources show rapid, regular and stable activity. The frequency spectrum obtained from sources maintaining AF usually shows a single frequency peak with a narrow morphological base [85]. Moreover, Takahashi *et* *al* [50], in a study with 25 patients, associated high OI values with sources that maintain the arrhythmia. Due to the high frequency of the ectopic focus, the rest of the atria cannot follow a 1∶1 activation, therefore, the conduction block results in a reduction of DF in remote areas of the focal source, as suggested by different authors [16], [19], [20]. Additionally, several studies in which AF is maintained by a focal source in the LA have reported higher DF values in the LA than in the RA [16], [18], [25].

Our results show EGM with potentials that display a pronounced negative deflection when the curved front passes on the recorded points. Jacquemet *et* *al* [13] also obtained potentials with a negative deflection much larger than the positive deflection in the presence of curved fronts.

In areas of the atria away from the ectopic focus, multiple reentrant waves that collided and fragmented were observed, which produced irregular and polymorphic EGM with single, double and CFAE; a hallmark of fibrillatory conduction [10], [15], [77]. Indeed, double potentials are related with wave fragmentations and conduction block and CFAE are related with pivot points, as previously mentioned. Finally, CL variations are reflected in the spectrum as multiple frequency peaks and low OI values, indicating an irregular conduction pattern with high frequency variability.

### Limitations of the study

Some of the limitations of this study include:

The Nygren model, despite being a fairly complex and detailed model, has some limitations. It lacks a detailed dynamic intracellular Ca^2+^ cycling, which can play a relevant role in some cardiac arrhythmias. Several electrophysiologically detailed human atrial cell models have been developed. Although all of them resemble the AP of human atria, they have different morphologies (see [86] for review). We used the modifications on I_K1_, I_CaL_ and I_t_ reported by Zhang *et* *al* [37] to obtain electrically remodeled atrial cells models. In that work, the authors modified two atrial cellular models (Nygren *et* *al* [35] and Courtemanche *et* *al* [87]) and observed that the remodeling conditions (called in this work AF-1) provoked similar APD_90_ reductions (62% versus 68%). In another study, Sánchez *et* *al* [88] compared the results of two atrial cell models (Courtemanche *et* *al* [87] and Maleckar *et* *al* [89]) using slightly different remodeling conditions. They observed that the APD_90_ reduction was also similar in both models. Even more, although the restitution curve in control presented some differences between the cellular models, under remodeling conditions both restitution curves flattened and were qualitatively very similar. They concluded that their study of AF dynamics was independent of the model used. The aforementioned results suggest that the main findings of our work are also independent of the cellular model used.

The remodeling conditions used in our study are based on the experimental work of Bosh et al [7]. However, another experimental study [6] has reported shorter reductions of APD during AF (around 50%) and several computational studies [37], [88], [90] have introduced the effect of electrical remodeling mainly using quantitatively different modifications in the same channels. These different remodeling conditions may produce different patterns of atrial arrhythmias and requires further investigation. Another limitation is that the heterogeneity of our model is based on canine atria data [36]. New experimental data about the effect of atrial electrical remodeling on the anatomical structures of human atria would allow more realistic simulations of AF dynamic under chronic AF. Although the used remodeling conditions can reproduce the action potential phenotype observed in patients with permanent AF, electrical remodeling is not the only process accompanying permanent AF. Indeed, many of these patients have significant structural remodeling with fibrosis, which contributes to short APD [91], [92] and increases the complexity of the arrhythmia [93].

Our 3D anatomical model of human atria does not take into account the real thickness of the atrial walls. Several studies have shown very complex fiber structures in areas of human atria [2], [27], [30], [31]. Our results were obtained using a specific virtual atria model. Although our model includes a great number of anatomical and morphological details, it corresponds to a particular set of parameters (electrophysiology, anatomy, fiber direction, anisotropy and heterogeneity, among others). For example, our model includes four pulmonary veins, which is the most common anatomical structure in humans though other patterns have been observed [94]. In addition, although there are also inter-subject differences in fiber orientation, we have tried to model the most common fiber orientation observed experimentally for the different parts of the atrial model. The influence of aging on the anatomy of the atria is also relevant [95]. Therefore, more comprehensive results could be obtained when considering inter-subject variability. Indeed, Romero *et* *al* [96] analyzed the impact of the ionic current variability in line with biological inter-subject differences, on the APD_90_ in a model of human ventricular cells. Recently, a semi-automatic method to incorporate atrial anisotropy, heterogeneities and fiber orientation into patient specific models has been developed [97]. Although the fiber orientation was also based on data from literature and from different human atria preparations, the specific anatomy of patients is reproduced in 3D models using data from CT and MRI. This method could be used to further extend this study for personalized anatomical models.

Finally, the mesh used in the present study is relatively coarse. It comprises of 100554 nodes with a spatial resolution ranging from 300 to 700 μm (530 μm in average). Although this discretization is similar to the used in previous simulation studies of AF [26], [51] recent works have used higher resolutions. Aslanidi *et* *al* [62] used a spatial resolution of 330×330×300 μm^3^ and showed that decreasing the space step form 330 μm to 250 μm resulted in very small (<3%) changes of the conduction velocity of AP. Although a higher spatial resolution could slightly affect to AF dynamics observed in our study, we would expect similar patterns of activation. Moreover, these changes would not affect the relationship between atrial activation patterns and simulated EGMs.

## Conclusions

We have developed a realistic 3D model of human atria with a very detailed fiber orientation, real anisotropy and electrophysiological properties. Additionally, it includes all main interatrial connections. To our knowledge, this model is one of the most complete human atrial models developed.

In our study using an electrically remodeled atria model, a transient focus induced atrial flutter, reentrant tachycardias and AF; whereas a continuous ectopic focus provoked AF in all of the tested sites.

Our model can reproduce the stability and regularity that cause arrhythmias as AFL, reentrant tachycardia and areas with focal activity. This electrical activity is reflected in EGM with single potentials and spectra with a narrow frequency peak and high OI values. Our model can also reproduce characteristic patterns of AF, which were maintained by multiple reentrant waves, rotors, fragmentation and wave collision, which are reflected in polymorphic EGM and spectra with multiple frequency peaks and low OI values. Interestingly, EGM potentials with only a negative deflection are related to spiral and curved wavefronts that pass and move away. In addition, potentials with a much greater positive deflection are related with wave collisions. Moreover, double potentials are related with either wave fragmentations or blocking lines, while CFAE are related to pivot points.

Finally, this is the first work that uses a 3D human atrial to investigate the relationship between different atrial arrhythmic propagation patterns and the EGM observed at more than 43000 points on the atrial surface.

## Supporting Information

Video S1 **Atrial Flutter** (**supplements** **Fig. 5A** **in the manuscript**)**.** Simulation of an AFL episode, triggered by six ectopic beats applied at the isthmus of the RA (near the CS), and maintained by a macroreentry rotating counterclockwise around the tricuspid annulus.
(AVI)

Video S2 **Reentrant tachycardia (supplements** **Fig. 5B** **in the manuscript).** Simulation of reentrant tachycardia episode, triggered by six ectopic beats applied at the middle of the CT, and maintained by a reentry anchored to the RA, moving between the center and the top areas of the CT.
(AVI)

Video S3 **Atrial fibrillation induced by a transitory focus** (**supplements** **Fig. 6A** **in the manuscript**). Simulation of AF episode, triggered by six ectopic beats applied in the ostium of the RPV, and maintained by multiple reentrant waves. Rotors, figure of eight reentries, macrorreentries, fragmentations and wavefront collisions can be observed in both atria.
(AVI)

Video S4 **Atrial fibrillation induced by a continuous focus** (**supplements** **Fig.** **6B** **in the manuscript**). Simulation of focal AF episode, triggered by a continuous ectopic focus applied in the ostium of the RPV. Multiple reentrant waves are observed in the RA and in the superior and anterior walls of LA. Posterior wall near to RPV show stable activity at high frequency due to the wavefront generated by the ectopic focus.
(AVI)

## References

[1] Wolf P, Benjamin E, Belanger A, Kannel W, Levy D, et al (1996) Secular trends in the prevalence of atrial fibrillation: The Framingham study. Am Heart J. 113: 790–796. 10.1016/s0002-8703(96)90288-4 8721656

[2] Ho SY, Anderson RH, Sanchez-Quintana D (2002) Atrial structure and fibres: morphologic bases of atrial conduction. Cardiovasc Res. 54(2): 325–336. 10.1016/s0008-6363(02)00226-2 12062338

[3] Lesh MD, Kalman JM, Olgin JE, Ellis WS (1996) The role of atrial anatomy in clinical atrial arrhythmias. J Electrocardiol. 29(1): 101–113. 10.1016/s0022-0736(96)80039-2 9238386

[4] Wilders R, Wagner MB, Golod DA, Kumar R, Wang YG, et al (2000) Effects of anisotropy on the development of cardiac arrhythmias associated with focal activity. Pflugers Arch. 441: 301–312. 10.1007/s004240000413 11211117

[5] Nattel S (2002) New ideas about atrial fibrillation 50 years on. Nature. 415(6868): 219–226. 10.1038/415219a 11805846

[6] Workman AJ, Kane AK, Rankin AC (2001) The contribution of ionic currents to changes in refractoriness of human atrial myocytes associated with chronic atrial fibrillation. Cardiovasc Res. 52(2): 226–235. 10.1016/s0008-6363(01)00380-7 11684070

[7] Bosch RF, Zeng X, Grammer JB, Popovic CM, Mewis C, et al (1999) Ionic mechanisms of electrical remodeling in human atrial fibrillation. Cardiovasc Res. 44: 121–231. 10.1016/s0008-6363(99)00178-9 10615396

[8] Wijffels MCEF, Kirchhof CJHJ, Dorland R, Allessie MA (1995) Atrial-fibrillation begets atrial-fibrillation – a study in awake chronically instrumented goats. Circulation. 92(7): 1954–1968. 10.1161/01.cir.92.7.1954 7671380

[9] Haissaguerre M, Jais P, Shah DC, Takahashi A, Hocini M, et al (1998) Spontaneous initiation of atrial fibrillation by ectopic beats originating in the pulmonary veins. N Engl J Med. 339(10): 659–666. 10.1056/NEJM199809033391003 9725923

[10] Mandapati R, Skanes A, Chen J, Berenfeld O, Jalife J (2000) Stable microreentrant sources as a mechanism of atrial fibrillation in the isolated sheep heart. Circulation. 101(2): 194–199. 10.1161/01.cir.101.2.194 10637208

[11] Allessie MA, Bonke FI, Schopman FJ (1977) Circus movement in rabbit atrial muscle as a mechanism of tachycardia. III. The “leading circle” concept: a new model of circus movement in cardiac tissue without the involvement of an anatomical obstacle. Circ Res. 41(1): 9–18. 10.1161/01.res.41.1.9 862147

[12] Moe GK (1962) On the multiple wavelet hypothesis of atrial fibrillation. Arch Int Pharmacodyn. 140: 183–188.

[13] Jacquemet V, Virag N, Ihara Z, Dang L, Blanc O, et al (2003) Study of unipolar electrogram morphology in a computer model of atrial fibrillation. J Cardiovasc Electrophysiol. 14: S172–S179. 10.1046/j.1540.8167.90308.x 14760921

[14] Zlochiver S, Yamazaki M, Kalifa J, Berenfeld O (2008) Rotor meandering contributes to irregularity in electrograms during atrial fibrillation. Heart Rhythm. 5(6): 846–854. 10.1016/j.hrthm.2008.03.010 PMC3079377 18534369

[15] Konings KT, Smeets JL, Penn OC, Wellens HJ, Allessie MA (1997) Configuration of unipolar atrial electrograms during electrically induced atrial fibrillation in humans. Circulation. 95(5): 1231–1241. 10.1161/01.cir.95.5.1231 9054854

[16] Lin YJ, Tai CT, Chen SA (2006) Can mapping and ablation of atrial fibrillation be guided by frequency analysis of fibrillatory waves? J Cardiovasc Electrophysiol. 17(3): S44–S49. 10.1046/j.1540-8167.2004.04274.x 15333086

[17] Kalifa J, Tanaka K, Zaitsev AV, Warren M, Vaidyanathan R, et al (2006) Mechanisms of wave fractionation at boundaries of high-frequency excitation in the posterior left atrium of the isolated sheep heart during atrial fibrillation. Circulation. 113(5): 626–633. 10.1161/CIRCULATIONAHA.105.575340 16461834

[18] Berenfeld O, Mandapati R, Dixit S, Skanes AC, Chen J, et al (2000) Spatially distributed dominant excitation frequencies reveal hidden organization in atrial fibrillation in the Langendorff-perfused sheep heart. J Cardiovasc Electrophysiol. 11(8): 869–879. 10.1111/j.1540-8167.2000.tb00066.x 10969749

[19] Sanders P, Berenfeld O, Hocini M, Jais P, Vaidyanathan R, et al (2005) Spectral analysis identifies sites of high-frequency activity maintaining atrial fibrillation in humans. Circulation. 112(6): 789–797. 10.1161/CIRCULATIONAHA.104.517011 16061740

[20] Lazar S, Dixit S, Marchlinski FE, Callans DJ, Gerstenfeld EP (2004) Presence of left-to-right atrial frequency gradient in paroxysmal but not persistent atrial fibrillation in humans. Circulation. 110(20): 3181–3186. 10.1161/01.CIR.0000147279.91094.5E 15533867

[21] Sanders P, Nalliah CJ, Dubois R, Takahashi Y, Hocini M, et al (2006) Frequency mapping of the pulmonary veins in paroxysmal versus permanent atrial fibrillation. J Cardiovasc Electrophysiol. 17(9): 965–972. 10.1111/j.1540-8167.2006.00546.x 16948740

[22] Everett TH, Kok LC, Vaughn RH, Moorman JR, Haines DE (2001) Frequency domain algorithm for quantifying atrial fibrillation organization to increase defibrillation efficacy. IEEE Trans Biomed Eng. 48(9): 969–978. 10.1109/10.942586 11534845

[23] Everett TH, Wilson EE, Verheule S, Guerra JM, Foreman S, et al (2006) Structural atrial remodeling alters the substrate and spatiotemporal organization of atrial fibrillation: a comparison in canine models of structural and electrical atrial remodeling. Am J Physiol Heart Circ Physiol. 291(6): H2911–H2923. 10.1152/ajpheart.01128.2005. PMC2062526 16877548

[24] Skanes AC, Mandapati R, Berenfeld O, Davidenko JM, Jalife J (1998) Spatiotemporal periodicity during atrial fibrillation in the isolated sheep heart. Circulation. 98(12): 1236–1248. 10.1161/01.cir.98.12.1236 9743516

[25] Mansour M, Mandapati R, Berenfeld O, Chen J, Samie FH, et al (2001) Left-to-right gradient of atrial frequencies during acute atrial fibrillation in the isolated sheep heart. Circulation. 103(21): 2631–2636. 10.1161/01.cir.103.21.2631 11382735

[26] Harrild D, Henriquez C (2000) A computer model of normal conduction in the human atria. Circ Res. 87(7): E25–E36. 10.1161/01.res.87.7.e25 11009627

[27] Ho SY, Sanchez-Quintana D, Cabrera JA, Anderson RH (1999) Anatomy of the left atrium: implications for radiofrequency ablation of atrial fibrillation. J Cardiovasc Electrophysiol. 10(11): 1525–1533. 10.1111/j.1540-8167.1999.tb00211.x 10571372

[28] Cohen GI, White M, Sochowski RA, Klein AL, Bridge PD, et al (1995) Reference values for normal adult transesophageal echocardiographic measurements. J Am Soc Echocardiogr. 8(3): 221–230. 10.1016/s0894-7317(05)80031-8 7640014

[29] Ho SY, Sanchez-Quintana D, Anderson RH (1998) Can anatomy define electric pathways? In: International Workshop on Computer Simulation and Experimental Assessment of Electrical Cardiac Function, Lausanne, Switzerland. 77–86.

[30] Nathan H, Eliakim M (1966) The Junction Between the Left Atrium and the Pulmonary Veins: An Anatomic Study of Human Hearts. Circulation. 34: 412–422. 10.1161/01.cir.34.3.412 5922708

[31] Ho S, Sanchez-Quintana D (2009) The importance of atrial structure and fibers. Clinical Anatomy. 22: 52–63. 10.1002/ca.20634 18470938

[32] Tobón C (2009) Evaluación de factores que provocan fibrilación auricular y de su tratamiento mediante técnicas quirúrgicas. Estudio de simulación. Master Thesis Universitat Politècnica de València.

[33] Ruiz C (2010) Estudio de la vulnerabilidad a reentradas a través de modelos matemáticos y simulación de la aurícula humana. Doctoral Thesis Universitat Politècnica de València.

[34] Tobón C (2010) Modelización y evaluación de factores que favorecen las arritmias auriculares y su tratamiento mediante técnicas quirúrgicas. Estudio de simulación. Doctoral Thesis Universitat Politècnica de València.

[35] Nygren A, Fiset C, Firek L, Clark JW, Lindblad DS, et al (1998) Mathematical model of an adult human atrial cell: the role of K+ currents in repolarization. Circ Res. 82(1): 63–81. 10.1161/01.res.82.1.63 9440706

[36] Feng J, Yue L, Wang Z, Nattel S (1998) Ionic mechanisms of regional action potential heterogeneity in the canine right atrium. Circ Res. 83(5): 541–551. 10.1161/01.res.83.5.541 9734477

[37] Zhang H, Garratt CJ, Zhu J, Holden AV (2005) Role of up-regulation of IK1 in action potential shortening associated with atrial fibrillation in humans. Cardiovasc Res. 66: 493–502. 10.1016/j.cardiores.2005.01.020 15914114

[38] Clayton RH, Holden AV (2004) Propagation of normal beats and re-entry in a computational model of ventricular cardiac tissue with regional differences in action potential shape and duration. Prog Biophys Mol Biol. 85: 473–499. 10.1016/j.pbiomolbio.2003.12.002 15142758

[39] Henriquez CS, Papazoglou AA (1996) Using computer models to understand the roles of tissue structure and membrane dynamics in arrhythmogenesis. Proceedings of the IEEE 84(3): 334–354.

[40] Rogers JM, McCulloch AD (1994) A collocation-Galerkin finite element model of cardiac action potential propagation. IEEE Trans Biomed Eng. 41: 743–757. 10.1109/10.310090 7927397

[41] Dolber PC, Spach MS (1989) Structure of canine Bachmann's bundle related to propagation of excitation. Am J Physiol. 257(5 Pt 2): H1446–H1457. 10.1152/ajpheart.1989.257.5.H1446 2589500

[42] Hayashi H, Lux RL, Wyatt RF, Burgess MJ, Abildskov JA (1982) Relation of canine atrial activation sequence to anatomic landmarks. Am J Physiol. 242(3): H421–H428. 10.1152/ajpheart.1982.242.3.H421 7065202

[43] Boineau JP, Canavan TE, Schuessler RB, Cain ME, Corr PB, et al (1988) Demonstration of a widely distributed atrial pacemaker complex in the human heart. Circulation. 77(6): 1221–1237. 10.1161/01.cir.77.6.1221 3370764

[44] Hansson A, Holm M, Blomstrom P, Johansson R, Luhrs C, et al (1998) Right atrial free wall conduction velocity and degree of anisotropy in patients with stable sinus rhythm studied during open heart surgery. Eur Heart J. 19(2): 293–300. 10.1053/euhj.1997.0742 9519324

[45] Arora R, Verheule S, Scott L, Navarrete A, Katari V, et al (2003) Arrhythmogenic substrate of the pulmonary veins assessed by high-resolution optical mapping. Circulation. 107(13): 1816–1821. 10.1161/01.CIR.0000058461.86339.7E PMC1995670 12665495

[46] Kleber AG, Rudy Y (2004) Basic mechanisms of cardiac impulse propagation and associated arrhythmias. Physiol Rev. 84(2): 431–488. 10.1152/physrev.00025.2003 15044680

[47] Gima K, Rudy Y (2002) Ionic current basis of electrocardiographic waveforms: a model study. Circ Res. 90: 889–896. 10.1161/01.res.0000016960.61087.86 PMC1847799 11988490

[48] Saiz J, Gomis-Tena J, Monserrat M, Ferrero JM, Cardona K, Chorro J (2011) Effects of the antiarrhythmic drug dofetilide on transmural dispersion of repolarization in ventriculum. A computer modelling study. IEEE Trans Biomed Eng. 58(1): 43–53. 10.1109/TBME.2010.2077292 20851784

[49] Nademanee K, McKenzie J, Kosar E, Schwab M, Sunsaneewitayakul B, et al (2004) A new approach for catheter ablation of atrial fibrillation: mapping of the electrophysiologic substrate. J Am Coll Cardiol. 43: 2044–2053. 10.1016/j.jacc.2003.12.054 15172410

[50] Takahashi Y, Sanders P, Jais P, Hocini M, Dubois R, et al (2006) Organization of frequency spectra of atrial fibrillation: relevance to radiofrequency catheter ablation. J Cardiovasc Electrophysiol. 17(4): 382–388. 10.1111/j.1540-8167.2005.00414.x 16643359

[51] Gong Y, Xie F, Stein KM, Garfinkel A, Culianu CA, et al (2007) Mechanism underlying initiation of paroxysmal atrial flutter/atrial fibrillation by ectopic foci: a simulation study. Circulation. 115: 2094–2102. 10.1161/CIRCULATIONAHA.106.656504 17420354

[52] Heidenreich EA, Ferrero JM, Doblare M, Rodriguez JF (2010) Adaptive macro finite elements for the numerical solution of monodomain equations in cardiac electrophysiology. Ann Biomed Eng. 38: 2331–2345. 10.1007/s10439-010-9997-2 20238165

[53] Niederer SA, Kerfoot E, Benson AP, Bernabeu MO, Bernus O, et al (2011) Verification of cardiac tissue electrophysiology simulators using an N-version benchmark. Philos Transact A Math Phys Eng Sci. 369: 4331–4351. 10.1098/rsta.2011.0139 PMC3263775 21969679

[54] Strang G (1968) On the construction and comparison of difference schemes. SIAM J. Numer Anal. 5(3): 506–517.

[55] Kholova I, Kautzner J (2004) Morphology of atrial myocardial extensions into human caval veins: a postmortem study in patients with and without atrial fibrillation. Circulation. 110: 483–488. 10.1161/01.CIR.0000137117.87589.88 15277325

[56] Cabrera JA, Sanchez-Quintana D, Ho SY, Medina A, Anderson RH (1998) The architecture of the atrial musculature between the orifice of the inferior caval vein and the tricuspid valve: The anatomy of the isthmus. J Cardiovasc Electrophysiol. 9: 1186–1195. 10.1111/j.1540-8167.1998.tb00091.x 9835263

[57] Weigner MJ, Katz SE, Douglas PS, Manning WJ (1999) Left atrial appendage anatomy and function: short term response to sustained atrial fibrillation. Heart. 82: 555–558. 10.1136/hrt.82.5.555 PMC1760787 10525507

[58] Wang K, Ho SY, Gibson DG, Anderson RH (1995) Architecture of Atrial Musculature in Humans. British Heart J. 73: 559–565. 10.1136/hrt.73.6.559 PMC483920 7626357

[59] Lemery R, Guiraudon G, Veinot JP (2003) Anatomic description of Bachmann's bundle and its relation to the atrial septum. Am J Cardiol. 91(12): 1482–1485. 10.1016/s0002-9149(03)00405-3 12804741

[60] Sanchez-Quintana D, Anderson RH, Cabrera JA, Climent V, Martin R, et al (2002) The terminal crest: morphological features relevant to electrophysiology. Heart. 88(4): 406–411. 10.1136/heart.88.4.406 PMC1767383 12231604

[61] Seemann G, Hoper C, Sachse FB, Dossel O, Holden AV, et al (2006) Heterogeneous three-dimensional anatomical and electrophysiological model of human atria. Philos Transact A Math Phys Eng Sci. 364(1843): 1465–1481. 10.1098/rsta.2006.1781 16766355

[62] Aslanidi OV, Colman MA, Stott J, Dobrzynski H, Boyett MR, et al (2011) 3D virtual human atria: A computational platform for studying clinical atrial fibrillation. Prog Biophys Mol Biol. 107(1): 156–168. 10.1016/j.pbiomolbio.2011.06.011 PMC3211061 21762716

[63] Krogh-Madsen T, Abbott GW, Christini DJ (2012) Effects of Electrical and Structural Remodeling on Atrial Fibrillation Maintenance: A Simulation Study. PLoS Comput Biol. 8(2): e1002390. 10.1371/journal.pcbi.1002390 PMC3285569 22383869

[64] Zhao J, Butters TD, Zhang H, Pullan AJ, LeGrice IJ, et al (2012) An image-based model of atrial muscular architecture: effects of structural anisotropy on electrical activation. Circ Arrhythm Electrophysiol. 5: 361–370. 10.1161/CIRCEP.111.967950 22423141

[65] Antz M, Otomo K, Arruda M, Scherlag BJ, Pitha J, et al (1998) Electrical conduction between the right atrium and the left atrium via the musculature of the coronary sinus. Circulation. 98(17): 1790–1795. 10.1161/01.cir.98.17.1790 9788835

[66] Lemery R, Birnie D, Tang AS, Green M, Gollob M, et al (2007) Normal atrial activation and voltage during sinus rhythm in the human heart: an endocardial and epicardial mapping study in patients with a history of atrial fibrillation. J Cardiovasc Electrophysiol. 18(4): 402–408. 10.1111/j.1540-8167.2007.00762.x 17394455

[67] Canavan TE, Schuessler RB, Cain ME, Lindsay BD, Boineau JP, et al (1988) Computerized global electrophysiological mapping of the atrium in a patient with multiple supraventricular tachyarrhythmias. Ann Thorac Surg. 46(2): 232–235. 10.1016/s0003-4975(10)65904-8 3401083

[68] Durrer D, van Dam RT, Freud GE, Janse MJ, Meijler FL, et al (1970) Total excitation of the isolated human heart. Circulation. 41(6): 899–912. 10.1161/01.cir.41.6.899 5482907

[69] Franz MR, Karasik PL, Li C, Moubarak J, Chavez M (1997) Electrical remodeling of the human atrium: similar effects in patients with chronic atrial fibrillation and atrial flutter. J Am Coll Cardiol. 30(7): 1785–1792. 10.1016/s0735-1097(97)00385-9 9385908

[70] Gaspo R, Bosch RF, Talajic M, Nattel S (1997) Functional mechanisms underlying tachycardia-induced sustained atrial fibrillation in a chronic dog model. Circulation. 96(11): 4027–4035. 10.1161/01.cir.96.11.4027 9403628

[71] Chen SA, Hsieh MH, Tai CT, Tsai CF, Prakash VS, et al (1999) Initiation of atrial fibrillation by ectopic beats originating from the pulmonary veins: electrophysiological characteristics, pharmacological responses, and effects of radiofrequency ablation. Circulation. 100(18): 1879–1886. 10.1161/01.cir.100.18.1879 10545432

[72] Lin WS, Tai CT, Hsieh MH, Tsai CF, Lin YK, et al (2003) Catheter ablation of paroxysmal atrial fibrillation initiated by non-pulmonary vein ectopy. Circulation. 107(25): 3176–3183. 10.1161/01.CIR.0000074206.52056.2D 12821558

[73] Saoudi N, Cosío F, Waldo A, Chen SA, Iesaka Y, et al (2001) A classification of atrial flutter and regular atrial tachycardia according to electrophysiological mechanisms and anatomical bases. Eur Heart J. 22: 1162–1182. 10.1053/euhj.2001.2658 11440490

[74] Friedman PA, Luria D, Fenton AM, Munger TM, Jahangir A, et al (2000) Global right atrial mapping of human atrial flutter: the presence of posteromedial (sinus venosa region) functional block and double potentials: a study in biplane fluoroscopy and intracardiac echocardiography. Circulation. 101(13): 1568–1577. 10.1161/01.cir.101.13.1568 10747351

[75] Derejko P, Bodalski R, Szumowski LJ, Kozlowski D, Urbanek P, et al (2010) Relationship between cycle length of typical atrial flutter and double potential interval after achievement of complete isthmus block. Pacing Clin Electrophysiol. 33(12): 1518–1527. 10.1111/j.1540-8159.2010.02847.x 20663068

[76] Grimm RA, Chandra S, Klein AL, Stewart WJ, Black IW, et al (1996) Characterization of left atrial appendage Doppler flow in atrial fibrillation and flutter by Fourier analysis. Am Heart J. 132(2 Pt 1): 286–296. 10.1016/s0002-8703(96)90424-x 8701889

[77] Ryu K, Sahadevan J, Khrestian CM, Stambler BS, Waldo AL (2006) Use of fast fourier transform analysis of atrial electrograms for rapid characterization of atrial activation-implications for delineating possible mechanisms of atrial tachyarrhythmias. J Cardiovasc Electrophysiol. 17(2): 198–206. 10.1111/j.1540-8167.2005.00320.x 16533258

[78] Olshansky B, Wilber DJ, Hariman RJ (1992) Atrial flutter – update on the mechanism and treatment. Pacing Clin Electrophysiol. 15(12): 2308–2335. 10.1111/j.1540-8159.1992.tb04174.x 1282252

[79] Kalman JM, Olgin JE, Karch MR, Hamdan M, Lee RJ, et al (1998) “Cristal tachycardias”: origin of right atrial tachycardias from the crista terminalis identified by intracardiac echocardiography. J Am Coll Cardiol. 31(2): 451–459. 10.1016/s0735-1097(97)00492-0 9462592

[80] Becker R, Bauer A, Metz S, Kinscherf R, Senges JC, et al (2001) Intercaval block in normal canine hearts: role of the terminal crest. Circulation. 103(20): 2521–2526. 10.1161/01.cir.103.20.2521 11369695

[81] Chorro FJ, Ferrero A, Canoves J, Mainar L, Porres JC, et al (2003) Significance of the morphological patterns of electrograms recorded during ventricular fibrillation: an experimental study. Pacing Clin Electrophysiol. 26(5): 1262–1269. 10.1046/j.1460-9592.2003.t01-1-00178.x 12765456

[82] Berenfeld O, Zaitsev AV, Mironov SF, Pertsov AM, Jalife J (2002) Frequency-dependent breakdown of wave propagation into fibrillatory conduction across the pectinate muscle network in the isolated sheep right atrium. Circ Res. 90: 1173–1180. 10.1161/01.res.0000022854.95998.5c 12065320

[83] Jalife J (2003) Rotors and spiral waves in atrial fibrillation. J Cardiovasc Electrophysiol. 14(7): 776–780. 10.1046/j.1540-8167.2003.03136.x 12930260

[84] Nademanee K, Lockwood E, Oketani N, Gidney B (2010) Catheter ablation of atrial fibrillation guided by complex fractionated atrial electrogram mapping of atrial fibrillation substrate. J Cardiology. 55: 1–12. 10.1016/j.jjcc.2009.11.002 20122543

[85] Sahadevan J, Ryu K, Peltz L, Khrestian CM, Stewart RW, et al (2004) Epicardial mapping of chronic atrial fibrillation in patients: preliminary observations. Circulation. 110(21): 3293–3299. 10.1161/01.CIR.0000147781.02738.13 15520305

[86] Dossel O, Krueger MW, Weber FM, Wilhelms M, Seemann G (2012) Computational modeling of the human atrial anatomy and electrophysiology. Med Biol Eng Comput. 50: 773–799. 10.1007/s11517-012-0924-6 22718317

[87] Courtemanche M, Ramirez RJ, Nattel S (1999) Ionic targets for drug therapy and atrial fibrillation-induced electrical remodeling: insights from a mathematical model. Cardiovasc Res. 42: 477–489. 10.1016/s0008-6363(99)00034-6 10533583

[88] Sanchez C, Corrias A, Bueno-Orovio A, Davies M, Swinton J, et al (2012) The Na+/K+ pump is an important modulator of refractoriness and rotor dynamics in human atrial tissue. Am J Physiol Heart Circ Physiol. 302: H1146–H1159. 10.1152/ajpheart.00668.2011 PMC3311461 22198174

[89] Maleckar MM, Greenstein JL, Giles WR, Trayanova NA (2009) K+ current changes account for the rate dependence os the action potential in the human atrial myocyte. Am J Physiol Heart Circ Physiol 297: H1398–H1410. 19633207 10.1152/ajpheart.00411.2009 PMC2770776

[90] Grandi E, Pandit SV, Voigt N, Workman AJ, Dobrev D, et al (2011) Human atrial action potential and Ca2+ model: sinus rhythm and chronic atrial fibrillation. Circ Res. 109(9): 1055–1066. 10.1161/CIRCRESAHA.111.253955 PMC3208665 21921263

[91] Maleckar MM, Greenstein JL, Giles WR, Trayanova NA (2009) Eletrotonic coupling beteen human atrial myocytes and fibroblasts aters myocyte excitability and repolarization. Biophys J. 97: 2179–2190. 10.1016/j.bpj.2009.07.054 PMC2764083 19843450

[92] Ashihara T, Haraguchi R, Nakazawa K, Namba T, Ikeda T, et al (2012) The role of fibroblasts in complex fractionated electrograms during persistent/permanent atrial fibrillation: implications for electrogram-based catheter ablation. Circ Res. 110: 275–284. 10.1161/CIRCRESAHA.111.255026 PMC3313658 22179057

[93] Burstein B, Nattel S (2008) Atrial Fibrosis: Mechanisms and Clinical Relevance in Atrial Fibrillation. J Am Coll Cardiol. 51: 802–809. 10.1016/j.jacc.2007.09.064 18294563

[94] Marom EM, Herndon JE, Kim YH, McAdams HP (2004) Variations in pulmonary venous drainage to the left atrium: implications for radiofrequency ablation. Radiology. 230: 824–829. 10.1148/radiol.2303030315 14739316

[95] Pan NH, Tsao HM, Chang NC, Chen YJ, Chen SA (2008) Aging dilates atrium and pulmonary veins: implications for the genesis of atrial fibrillation. Chest. 133: 190–196. 10.1378/chest.07-1769 18187745

[96] Romero L, Pueyo E, Fink M, Rodriguez B (2009) Impact of ionic current variability on human ventricular cellular electrophysiology. Am J Physiol Heart Circ Physiol. 297: H1436–1445. 10.1152/ajpheart.00263.2009 19648254

[97] Krueger MW, Schmidt V, Tobón C, Weber FM, Lorenz C, et al (2011) Modeling Atrial Fiber Orientation in Patient-Specific Geometries: A Semi-automatic Rule-Based Approach. Lecture Notes in Computer Science. 6666: 223–232.

